# A dataset of Roman Urdu text with spelling variations for sentence level sentiment analysis

**DOI:** 10.1016/j.dib.2024.111170

**Published:** 2024-11-23

**Authors:** Mudasar Ahmed Soomro, Rafia Naz Memon, Asghar Ali Chandio, Mehwish Leghari, Muhammad Hanif Soomro

**Affiliations:** a Department of Information Technology, Quaid-e-Awam University of Engineering, Science & Technology, Nawabshah, Pakistan; b Department of Software Engineering, Quaid-e-Awam University of Engineering, Science & Technology, Nawabshah, Pakistan; c Department of Artificial Intelligence, Quaid-e-Awam University of Engineering, Science & Technology, Nawabshah, Pakistan; d School of Engineering and Information Technology, The University of New South Wales, Australia; e Department of Data Science, Quaid-e-Awam University of Engineering, Science & Technology, Nawabshah, Pakistan; f Department of Information Technology, University of Sindh, Jamshoro, Pakistan

**Keywords:** Roman Urdu, Sentiment analysis of Roman Urdu, Roman Urdu with spelling variations, Machine learning for Roman Urdu sentiment analysis, BLSTM for Roman Urdu sentiment analysis

## Abstract

Roman Urdu text is very widespread on many websites. People mostly prefer to give their social comments or product reviews in Roman Urdu, and Roman Urdu is counted as non-standard language. The main reason for this is that there is no rule for word spellings within Roman Urdu words, so people create and post their own word spellings, like “2mro” is a nonstandard spelling for tomorrow. This paper aims to collect two Roman Urdu datasets: one is roman Urdu words with various spelling variations. This dataset contains 5244 Roman Urdu words, within which we have included variations in word spellings ranging from (one) to (five) different spellings for each word. The second dataset consists of Roman Urdu reviews, which were collected from (seven) different internet-based sources. This dataset contains multiclass reviews, namely “very positive,” “positive,” “very negative,” “negative,” and “neutral”, respectively. We gathered a total of 28,090 reviews. The sentiments of the reviews were made by the domain experts who were familiar with the Urdu language.

Specifications Table

**Table:** 

Subject	Computer Science
Specific subject area	Natural Language Processing, Sentiment Analysis, Roman Urdu
Type of data	Text Files
Data collection	Using Computer Application tool web scraper “ParseHub” for some website that was support this tool, If any website tool was not supporting, then the data was manually collected.
Data source location	Data source location was selected websites to gathering data included: “Daraz.pk”, “Facebook”,“Instagram”, “Pakistan.web”, “Whatmobile”, “UrduPoint”, “masala.tv” and “Hamariweb”.
Data accessibility	The two datasets presented in this article are freely available at: “https://data.mendeley.com/datasets/v5jfhsvtmd/5” 10.17632/v5jfhsvtmd.5

## 1 Value of the Data

• The roman Urdu words dataset is the first to be published for roman Urdu with five spelling variations of each word.
• The roman Urdu review dataset is the first to be published that contained unique roman Urdu word spellings with maximum number of variations.
• The dataset will be useful to help research community to develop sentiment analysis systems for Urdu or related languages such as Persian and Sindhi.
• Roman Urdu script is widely used for social content communication and observer that people feel comfortable by most people in South Asia [1]. Major newspaper publishers are now publishing manually generated roman Urdu versions of their newspapers, and this dataset will help in the development of real-world application and analysis.

## 2 Background

Roman Urdu writing system is used by Urdu speakers, where English letters are used for roman Urdu. The widespread use of roman Urdu often reflects various situations differently, such as Urdu speakers creating their own spellings for certain words, leading to spelling variations commonly appearing in roman Urdu. These spelling variations pose significant challenge in natural language processing tasks like spell checking, text classification, and information retrieval.

Existing research studies have shown that extensive work has been done on Roman Urdu sentiment analysis and several Roman Urdu datasets are publicly available. A research study in [2] was conducted on unsupervised lexical normalization of Roman Urdu, while another study focused on the lexical variation in Roman Urdu sentiment analysis [3]. In these two studies, the authors of the first study did not share the dataset, whereas the second shared the dataset, which comprised Roman Urdu reviews. However, there is still no dataset available that includes Roman Urdu words with corresponding spelling variations up to five or more words. A dataset containing Roman Urdu words with spelling variations would be extremely useful for the sentiment analysis and language translation tasks. If people write their comments with different spelling variations of the same word, may be the language translation system may not be able to recognize the same word. In this way, if the same word with any spelling variation is first changed to its standard spelling, then the language translation systems can recognize it and translate into another language. This will also help in automatic grammar correction. This dataset typically includes a wide range of commonly used Roman Urdu words along with their five different spelling variations.

For Roman Urdu sentiment analysis, a research study in [4] collected a total of 26,824 user reviews from DarazPK, and constructed a dataset known as the Roman Urdu E-commerce Dataset (RUECD). Another dataset [5], was collected from six different domains with total number of 11,000 reviews with binary classification. Furthermore, the third dataset collected by [6], included 3241 annotated sentiments. The dataset in [7] contained 10,021 reviews, denoted as RUSA-19, using the source from 566 online threads across various domains such as Sports, Software, Food & Recipes, Drama, and Politics. In the context of existing research, many researchers have highlighted the significant issue of spelling variations in Roman Urdu, posing challenges in sentiment analysis. To address this problem the collected datasets consists of Roman Urdu reviews. The first dataset contains larger number of roman Urdu reviews and within these reviews, spelling variations have been eliminated by replacing unique roman Urdu words, so that the problem of spelling variation may not be encountered in sentiment analysis. The purpose of creating these datasets is to facilitate research areas such as spelling correction algorithms, applications in sentiment analysis, customer feedback analysis, and language translation from roman Urdu to English, and language understanding in roman Urdu text. This dataset can serve as a valuable resource for market research, brand monitoring, and customer engagement strategies in Urdu speaking communities.

## 3 Data Description

### 3.1 Data collection Roman Urdu words with spelling variations

The first dataset was created by collecting 20,232 Roman Urdu reviews and stored in an Excel file. Every review obtained was tokenized^1^ to separate every word, after that we used manual method to find every roman Urdu word and stored it in another Excel file. It was observed that there were many roman Urdu words with two to five spelling variation. Each Roman Urdu word was searched and stored in a separate Excel file, and when any word with the same meaning but different spellings was found, it was stored in the respective column with its equivalent word. We had set a limitation that if a word had more than (five) spellings, we would only consider up to (five) spelling variations and save those five spellings accordingly. For creating the dataset, we used (seven) Excel's columns. Among them, (five) columns were named “Var-1”, “Var-2”, “Var-3”, “Var-4”, and “Var-5”, where the different spellings of the Roman Urdu word were saved. In the (sixth) column labelled as “Common,” we have placed the word that has been used most frequently in the entire dataset, regardless of its spelling variations. Like as per instructions, in the “Common” column, we have placed the word that has been used most frequently in the dataset. For example, if the word “acha” (اچھا) (“Ok” in English) was searched, with spellings “a6a” occurring 70 times, “axha” occurring 60 times, and “acha” occurring 150 times, then we would place “acha” in the “Common” column since it has been used most frequently in the given spelling. We have put the English translations of the Roman Urdu words in the last column. Out of the 20,232 reviews, when the reviews were tokenized and all Roman Urdu words were searched, there are a total of 5244 Roman Urdu words searched. However, when considering variations in spellings within the dataset, there are a total of 19,527 Roman Urdu words present as shown in Table 1. Within the searching process 1026 words are with (five) spelling variations, 2008 words with (four) spelling variations, 1987 words with (three) spelling variations, 181 words with (two) spelling variations, and 41 words with only one and unique word spelling.

**Table 1:** Details of Roman Urdu words with spelling variations.

RU words with spelling variation	Number RU words	Total words
RU words with 1 (One) Spellings	42×1	42
RU words with 2 (Two) Spellings	181×2	362
RU words with 3 (Three) Spellings	1987×3	5961
RU words with 4 (Four) Spellings	2008×4	8032
RU words with 5 (Five) Spellings	1026×5	5130
**Total Number of words**	**5244**	**19,527**

### 3.2 Data collection Roman Urdu reviews

After completing first dataset, we gathered another dataset that was collected from the most engaged sites in South Asia.^2^ While collecting text data, we used web scraper “ParseHub,” Python program [8]. The text data was manually collected as well. Within text scraping, we got four types of reviews: Roman Urdu reviews, reviews in English, pure Urdu reviews, and reviews that were with a mix of English and Roman Urdu words. We carefully examined all the reviews that were stored in the Excel file. Since our goal was to create a Roman Urdu dataset, we set a limitation that any review with at least 75 %of its review written in Roman Urdu would be included in the dataset. Additionally, each review should have a length of at least (hundred) words and contain a minimum of (two) Roman Urdu words.

### 3.3 Websites domains selection

Data was collected from seven distinct areas, each with its unique focus. These domains were related to different purposes like: Political affairs and hate speech, Dramas, Movies and Sports, entertainment, Music, Television Shows, Online Shopping Reviews, Food Recipes, Travel and Tourism for example, information gathered from “Daraz.pk” was related to online shopping reviews, whereas the data from “masala.tv” primarily focused on food-related reviews. We gathered data related to dramas, movies, and sports from Facebook, where we selected certain groups and pages relevant to movies, sports, dramas, and entertainment. From these sources, we extracted user reviews. In the end, total of 28,090 Roman Urdu reviews were collected from (seven) different domains.

### 3.4 Labelling data and the process of defining guidelines

When annotating the dataset, we took very carefully to review it in accordance with the guidelines established from existing work on annotation guidelines [9,10]. This enabled us to establish baseline guidelines for cases that were straightforward and unambiguous. Additionally, it provided a framework for ensuring consistency and accuracy throughout the annotation process. We incorporated input from individuals in selected domains by considering questions such as “Itna acha mobile hai muft maai lena chahiye ya nahi?” “ اتنا اچھا موبائل ہے مفت میں لینا چاہئے یا نہیں ؟ ” (“What a nice mobile, should it be purchased in a free or not?”) and “ham kab tak aisay logon ko vote dete rahay ge?” “ ہَم کب تک ایسے لوگوں کو ووٹ دیتے رہے گے ؟ ” (“How long will we keep voting for such people?”) to validate these types of reviews, a total of 831 reviews were identified. Three out of five annotators were assigned independently to annotate these reviews, The reviews we gave to annotators were finalized based on the majority of voting. We collected a total of 28,090 reviews, ensuring that were gathered and annotated according to the established guidelines. This approach was adopted to ensure the consistency and accuracy of sentiment annotations in the dataset. We annotated the dataset and defined criteria in a systematic way. The annotation procedures were established in two steps. First, we extensively studied existing work on annotation guidelines [[11], [12], [13]] and set baseline guidelines for straightforward cases.

The Roman Urdu review dataset was annotated according to the following guidelines:

**very-positive reviews:** Reviews expressing a high degree of satisfaction, happiness, or love annotated as “Very-positive”.

**positive reviews:** Reviews conveying simple positive feelings of happiness or satisfaction were annotated as “Positive”.

**Negative reviews**: Reviews expressing disappointment, sadness, or dissatisfaction were annotated as “Negative”.

**Very-Negative reviews**: Reviews containing profanity, insults, or extreme expressions of hatred were annotated as “Very-negative”.

**Neutral reviews**: Reviews that didn't contain explicit expressions of happiness, sadness, hatred, or love were classified as “Neutral”.

**Reviews that contain the combination positive and negative terms:** Some reviews contained both positive and negative expressions equally [14]. These were labelled as “Neutral”. Reviews where the majority of content was positive, but with few negative aspects, were annotated as “Positive”.

Table 2 provides an overview of sentiment distribution across various domains in the data collection process. Sentiments are categorized into five classes: Very Negative, Negative, Neutral, Positive, and Very Positive. The table presents the counts of comments within each sentiment category based on specific domains. When we have collected both datasets, one containing Roman Urdu words with spelling variations and the other containing Roman Urdu reviews, we observed that the Roman Urdu reviews dataset contained many Roman Urdu words with multiple spellings. Both of these reviews, “Aap kesy ho?” and “Ap ksy ho?” (“how are you?”), convey the same meaning and purpose, but the spellings of the words within both reviews are different.

**Table 2:** Details of the roman Urdu reviews dataset.

Domains	Very Positive	Positive	Neutral	Negative	Very Negative
Dramas, Movies and Sports	1052	1291	1124	906	589
Political affairs and hate speech	476	594	1388	1605	1577
Food recipe	552	689	754	671	451
News	747	943	826	750	412
Entertainment, Music, Television Shows	1280	1591	1021	1057	537
Online shopping	761	1047	1065	1007	727
Travel and Tourism	140	133	267	40	20
Total Number of annotated reviews	5008	6288	6445	6036	4313
Total number of reviews	28,090				

In last, our final objective was to remove spelling variations from the Roman Urdu review dataset, which was possible through the first dataset of Roman Urdu word spellings as explained prior in subsection 1.1. The dataset of Roman Urdu words with spelling variations, there is a word “aap” (آپ) (“you”) with 5 different spellings found: “aap”, “ap”, “app”, “aaap”, “aapp”. Among these, the most frequently used spelling was “ap”, which was assigned to the “Common” column. We created a dataset of unique word spellings in Roman Urdu reviews by identifying and replacing the words that we collected from the Roman Urdu words spelling variation dataset if there was a Roman Urdu word with (five) spelling variations, we searched for all five spellings in the Roman Urdu reviews dataset and replaced them with the spelling that we had selected as “common.” After finding and replacing all the words present in the Roman Urdu word dataset within our Roman Urdu reviews dataset, our Roman Urdu reviews dataset became significantly cleaner, with spelling variations largely eliminated. We successfully combined the two datasets and collected one dataset that containing Roman Urdu word spellings and the other containing Roman Urdu reviews.

## 4 Experimental Design, Materials and Methods

To assess the complexity and effectiveness of the datasets, two separate experiments were performed for the Roman Urdu word classification with spelling variations and Roman Urdu sentence level sentiment analysis. The experimental design, material and methods implemented for both experiments are discussed in the following subsections.

### 4.1 Roman Urdu word classification with spelling variations

When we constructed the first roman Urdu word spelling dataset, we employed supervised machine learning classification algorithms [15] for its validation. In the experimental phase of machine learning, the dataset was divided into 80 % for training and 20 % for testing for each classifier [16]. The results obtained are based on the performance of accuracy, precision, recall, and F1 score [17] as described in Table 3.

**Table 3:** Classification results using machine learning (%).

Classifier	Accuracy	Recall	F1 score	Precision
SVM	99	99	99	99
DT	98	98	98	98
NB	97	97	97	97
KNN	92	90	91	93

The machine learning classifier selected were Support Vector Machine, Logistic Regression, Decision Tree, Naïve Bayes, K-Nearest Neighbors, and Random Forest, for classifying the spelling variations. Upon evaluating their performance, we found that the Support Vector Machine classifier outperformed, while KNN's performance was the lowest as compared to others. Although, machine learning classifiers performed better on the Roman Urdu word dataset, however, for the Roman Urdu at sentence level, the performance of the classifiers was low. Only the SVM classifier obtained 81 %, while the other classifiers obtained less than 80 % accuracy.

### 4.2 Sentence level sentiment analysis of Roman Urdu

The second dataset was designed to support the evaluation of various deep learning model configurations, enabling researchers to explore and optimize model performance, which consists of Roman Urdu reviews at the sentence level [18]. We constructed Recurrent Neural Network-Long Short-Term Memory (RNN-LSTM), Recurrent Neural Network-bidirectional long short-term memory (RNN-BiLSTM), and Recurrent Convolutional Neural Network (RCNN) models at the sentence level as described in Table 4.

**Table 4:** Classification results with deep learning models (%).

Classifier	Precision	Recall	F1 score	Accuracy
RNN-LSTM	94	96	95	92
RNN-BiLSTM	94	95	97	92
R-CNN	96	95	96	93

Through experimentation, we observed that certain configurations of RNN-LSTM and RNN-BiLSTM models consistently achieved strong accuracy levels, while R-CNN models also demonstrated effective results. This dataset provides a robust foundation for assessing different RNN architectures, offering valuable insights for model comparison and optimization.

Table 5 describes the existing datasets and the experimental results obtained from them. In this study, two datasets were used: one is the RUSA-19 Corpus, which consists of 10,021 sentences, and the other is the Roman Urdu UCL dataset, containing 20,228 sentences. Experiments were conducted on both datasets using RCNN classifier with two types of models: one for binary classification and the other for tertiary classification. The accuracy achieved on the RUSA-19 Corpus was 74 for binary classification and 69 for tertiary classification. Similarly, the Roman Urdu UCL dataset achieved an accuracy of 72 for binary classification and 71 for tertiary classification [7]. When comparing the performance of both datasets with the proposed dataset, the proposed dataset demonstrated better performance than the existing datasets.

**Table 5:** RCNN evaluation results.

Dataset	Model	Precision	Recall	F1 score	Accuracy
RUSA-19 Corpus	Binary classification Tertiary classification	75 71	73 71	74 68	74 69
Roman Urdu UCL dataset	Binary classification Tertiary classification	73 69	72 73	72 69	72 71
Proposed DCNN	Multi-classification	96	95	96	93

## Limitations

Not applicable.

## Ethics Statement

To ensure confidence in the research, careful consideration was given to creating the dataset. It was ensured that no one's privacy was compromised in the compilation of the dataset. The reviews included in the dataset were obtained from sources where the public had posted them for public viewing, and no personal data of any individual was collected for inclusion in the dataset. *During the creation of the dataset, it was ensured that no individual's privacy would be highlighted.* It is important to mention that none of the reviews contain any personally identifiable information (PII)^3^.^4^ Ensuring the confidentiality of the user privacy and the protection of data integrity is crucial in establishing this fact. *All the data present in the dataset was already publicly available on public accessible websites. The websites we have selected are:* Daraz.pk,^5^ Facebook^6^^,^^7^”Instagram^8^^,^^9^”, “Pakistan.web^10^”, "Whatmobile^11^”, "UrduPoint^12^”, “masala.tv^13^” and “Hamariweb^14^”. *The web sources from where the data has been taken allow the data to be distributed publicly. The data has no copyright issue, as it is collected from the news and other web sources where the people post the reviews/comments for public access.*

## CRediT Author Statement

**Rafia Naz Memon:** Supervision, Reviewing and Editing. **Asghar Ali Chandio:** Co-Supervision, Conceptualization, Methodology. **Mehwish Leghari:** Data collection, annotation. **Muhammad Hanif Soomro:** Writing, Original draft preparation.

## Data Availability

Mendeley DataRoman Urdu Word Variations and Normalized Sentiment Review Dataset (RUWV-NSR) (Original data)..

## Footnotes

1 “https://support.microsoft.com/en-us/office/split-text-into-different-columns-with-the-convert-text-to-columns-wizard-30b14928-5550-41f5-97ca-7a3e9c363ed7” Last Visited on: 09-8-2023.

2 https://atmateen.com/pk/top-most-visited-websites-in-pakistan/ Last Visited on: 12-6-2023.

3 https://www.ibanet.org/ Last Visited on: 17-8-2023.

4 https://harvardlawreview.org/2014/12/data-mining-dog-sniffs-and-the-fourth-amendment/ Last Visited 17-8-2023.

5 https://www.daraz.pk/terms-conditions/ Last Visited 08-04-2024.

6 Public information on Facebook | Facebook Help Centre Last Visited 08-04-2024.

7 https://web.facebook.com/help/463983701520800?_rdc=1&_rdr Last Visited 08-04-2024.

8 https://help.instagram.com/581066165581870 Last Visited 08-04-2024.

9 https://help.instagram.com/155833707900388 Last Visited 10-04-2024.

10 https://www.pakistan.web.pk/help/privacy-policy/ Last Visited 10-04-2024.

11 https://www.whatmobile.com.pk/Privacy.php Last Visited 10-04-2024.

12 https://urdupoint.co/index.php/terms-and-conditions/ Last Visited 10-04-2024.

13 https://www.zaiqa.com/policy Last Visited 10-04-2024.

14 https://hamariweb.com/privacypolicy.aspx Last Visited 10-04-2024.
